# Analysis of pediatric absence epilepsy electroencephalograms using a mining-based association rule approach: A cross-sectional study based on realistic data

**DOI:** 10.1097/MD.0000000000050688

**Published:** 2026-09-18

**Authors:** Lijun Li, Lingxiang Ao, Lei Li, Li Wang, Kai Liu, Xiaomei Liu

**Affiliations:** a Neurophysiology Center, Kunming Children’s Hospital and Children’s Hospital of Kunming Medical University, Kunming, China; b Department of Quality and Safety, Kunming Children’s Hospital and Children’s Hospital of Kunming Medical University, Kunming, China; c Comprehensive Pediatrics, Kunming Children’s Hospital and Children’s Hospital of Kunming Medical University, Kunming, China.

**Keywords:** absence epilepsy, association rule mining, electroencephalography, seizure duration, spike-and-wave discharge

## Abstract

Association rule mining (ARM) is an interpretable data mining method that can identify co-occurrence patterns among predefined clinical features. This study applied ARM to clinically labeled EEG data from children with absence epilepsy to identify association rules linking abnormal EEG discharge morphology, scalp distribution, and absence seizure duration. Key association rules included polyspike-and-wave discharge → spike-and-wave discharge (support = 0.517, confidence = 1.000, lift = 1.034), spikes → spike-and-wave discharge (support = 0.510, confidence = 0.975, lift = 1.012), mesial temporal involvement → central involvement (support = 0.601, confidence = 0.968, lift = 1.528), and frontal pole involvement → frontal involvement (support = 0.629, confidence = 0.741, lift = 1.536). Frontal involvement combined with the 10–20-seconds seizure-duration category also showed high co-occurrence with frontal pole involvement ({F, 15.0 seconds} → {Fp}: support = 0.289, confidence = 1.000, lift = 1.561). These findings suggest that ARM can provide a structured and clinically readable summary of EEG co-occurrence patterns in pediatric absence epilepsy. However, the study was limited by its relatively small sample size, single-center design, expert manual labeling, binarized EEG representation, and lack of independent external validation. Therefore, the findings should be interpreted as exploratory and hypothesis-generating rather than externally validated clinical rules. Future multicenter studies with standardized EEG labeling, additional clinical variables, comparison with alternative analytical approaches, and independent validation datasets are needed to confirm the reproducibility and clinical applicability of these association rules.

## 1. Introduction

Absence epilepsy (AE) is a common epilepsy syndrome in children and is characterized by brief, recurrent episodes of impaired awareness.^[1]^ It is more common in females and typically begins between 4 and 12 years of age.^[2]^ AE may negatively affect learning, memory, behavior, and social functioning, thereby impairing quality of life.^[3]^ Electroencephalography (EEG) is central to the diagnosis of AE. Typical EEG findings include generalized, synchronous, and symmetric spike-and-wave discharges (SWDs), often at approximately 3 Hz.^[4]^ SWDs may vary in morphology, frequency, and distribution, including typical SWDs, polyspike-and-wave discharges, and rapid SWDs.^[5]^ In this study, scalp distribution was categorized according to electrode regions, including the frontal pole (Fp), frontal (F), central (C), mesial temporal (MT), parietal, and occipital (O) regions.^[6]^

Association rule mining (ARM) is a data mining method that seeks to identify the relationships between various elements within a given dataset.^[7]^ By placing the association rules between distinct brainwaves, ARM can be applied to EEG data analysis to unveil the intrinsic patterns and properties of brainwaves.^[8]^ Support, confidence, and lift are prevalent metrics employed in ARM to assess the frequency, dependability, and potency of association rules, respectively.^[9]^ EEG has emerged as a significant instrument in investigating epilepsy and various neurological disorders in recent decades.^[10]^ EEG enables researchers to discern patterns of brain electrical activity that occur during and between seizures by capturing electrical signals of brain activity.^[11]^ However, extracting valuable information from EEG data is difficult due to its diversity and complexity.^[12]^ To address this issue, the present study employed the data mining methodology known as ARM to examine EEG data.^[13]^ ARM is used in extensive datasets to identify noteworthy connections among objects.^[14]^ This approach possesses the capability to unveil latent, nonobvious correlations and patterns within the data, rendering it optimal for the examination of intricate and varied EEG data.^[15]^ We utilized this approach to analyze EEG data obtained from pediatric patients with AE to establish correlations among abnormal wave types, locations, and absence seizure duration.^[13]^

## 2. Materials and methods

### 2.1. Participants

We retrospectively reviewed a single-center cohort of pediatric patients with AE who attended the specialized epilepsy outpatient clinic at Kunming Children’s Hospital. The cohort included 153 patients (age 9 to 120 months; 89 female and 64 male), all of whom underwent video-EEG monitoring for at least 240 minutes. During monitoring, seizure frequency and duration were documented.

### 2.2. EEG monitoring protocol

Video-EEG monitoring was performed using a standard clinical V-EEG system, with electrodes placed according to the international 10 to 20 system. Each patient underwent at least 4 hours of monitoring covering wakefulness, sleep, and arousal after sleep deprivation. For children aged ≥4 years who were able to cooperate, activation procedures included eye opening/closure and hyperventilation for 3 minutes; if no epileptiform activity was induced, hyperventilation was extended to 5 minutes. During suspected clinical events, the examiner called the patient’s name to assess awareness.

### 2.3. Methods for processing data

To enhance the data’s quality and usability, the subsequent preprocessing, labeling, and binarization procedures were executed on the EEG data.

#### 2.3.1. Preprocessing

The EEG signal was decomposed into distinct frequency bands using a denoising method based on the wavelet transform. A suitable threshold was then chosen for each round, considering the signal-to-noise ratio, to eliminate noise components. Then, the denoised signal was reconstructed. Subsequently, the EEG signal was filtered using a Butterworth filter-based method to eradicate frequency components outside the range of 0.5 to 30 Hz, and divided into 10-second segments in preparation for subsequent labeling and analysis.

We used an expert knowledge-based labeling procedure. Each 10-second EEG segment was manually labeled by an experienced clinical EEG analyst according to a predefined coding scheme based on standard EEG terminology and the international 10 to 20 electrode system. SWD morphology was categorized as typical SWD (T), polyspike-and-wave discharge (P), spikes (S), and rapid SWD (R). The scalp distribution of SWDs was categorized into frontal pole (Fp), frontal (F), central (C), mesial temporal (MT), parietal, and occipital (O) regions. Absence seizure duration was categorized into 3 intervals: <10 seconds (L), 10 to 20 seconds (M), and >20 seconds (H).

#### 2.3.2. Labeling reliability and quality control

To improve labeling reliability, the final labels were checked for internal consistency against the predefined coding scheme and the original EEG annotations before binarization. Ambiguous or unclear segments were reexamined before final coding. Because of the retrospective design, formal inter-observer agreement statistics, such as Cohen’s κ, were not available. Therefore, the labeling procedure should be interpreted as expert-guided and quality-controlled, but not independently validated by formal inter-observer agreement analysis.

#### 2.3.3. Binarization

For ARM, each labeled 10-second EEG segment was transformed into a transaction-level binary vector. This binarization was necessary because the Apriori algorithm identifies frequent co-occurrence patterns among discrete items. The mapping was fixed as follows: bit1 = typical SWD, bit2 = polyspike-and-wave discharge, bit3 = spikes, bit4 = rapid SWD; bit5 = frontal pole, bit6 = frontal, bit7 = central, bit8 = mesial temporal, bit9 = parietal, bit10 = occipital; bit11 ≤ 10 seconds, bit12 = 10 to 20 seconds, and bit13 ≥ 20 seconds.

Seizure duration was discretized to make duration compatible with transaction-based rule mining and to reduce instability from overly granular event-level variation. This approach was designed to improve interpretability but inevitably reduced the continuous complexity of EEG and seizure-duration data. Each bit was set to 1 if the segment contained the corresponding feature, otherwise 0. For example, a segment containing all 4 SWD morphologies, with discharges localized to the frontal pole, frontal, and central regions, and with a 15-second absence seizure duration (M category) was encoded as 1111111000010. This binarized representation of each segment was used as the transaction input for ARM.

### 2.4. Association rule metrics

Typically, 3 essential concepts are employed: support, confidence, and lift. An overview of these concepts and their respective formulations is provided below.

#### 2.4.1. Magnitude of support

Support is calculated as the proportion of transactions, as a percentage of the total number of transactions, that comprise a specific itemset (or combination of itemsets). It determines the frequency with which a particular set of items appears in the dataset.

The calculation formula: Support(*A*) = count(*A*)/N, where count(*A*) is the number of segments containing itemset *A* and N is the total number of segments.


$$Support\left(X,Y\right)=P\left(XY\right)=\frac{numbers\left(XY\right)}{num\left(All\;Samples\right)}$$

#### 2.4.2. Level of confidence (math.)

Confidence is the conditional probability that another itemset B co-occurs with the occurrence of one itemset A. The correlation between two sets of items is quantified.

The calculation formula:


$$\begin{array}{c} Conf\;idence\left(X\to Y\right)=P(Y|X)=P\left(XY\right)/P\left(X\right) \end{array}$$

#### 2.4.3. Lift

Lift refers to the increase in the probability of another item set B occurring due to the occurrence of an item set A, relative to the initial probability of B occurring. With a threshold value of 1 (where 0 or less indicates a weaker relationship between A and B, and one or greater signifies a stronger one), lift indicates whether the correlation between A and B is softer or more potent than a random selection.

The calculation formula:


$$\begin{array}{c} Lift\left(X\to Y\right)=\frac{P(Y|X)}{P\left(Y\right)}=\frac{Conf\;idence\left(X\to Y\right)}{P\left(Y\right)} \end{array}$$

### 2.5. Mining association rules

We generated association rules using an Apriori algorithm-based method. Apriori was applied to the transaction matrix to enumerate frequent itemsets and then derive association rules, for which support, confidence, and lift were calculated. To reduce spurious rules driven by very low-frequency co-occurrences while retaining clinically interpretable directional patterns, we prespecified a minimum support of 0.05 and a minimum confidence of 0.50 for rule generation. Rules were further filtered by lift >1 and were prioritized primarily by lift, with confidence and support used to judge stability and practical relevance. Because the aim of this study was to extract interpretable co-occurrence rules from clinically labeled EEG features rather than to build a signal-level classifier, no direct machine learning or time-frequency benchmark model was constructed in the current analysis.

### 2.6. Validation procedure

**Figure 1.**
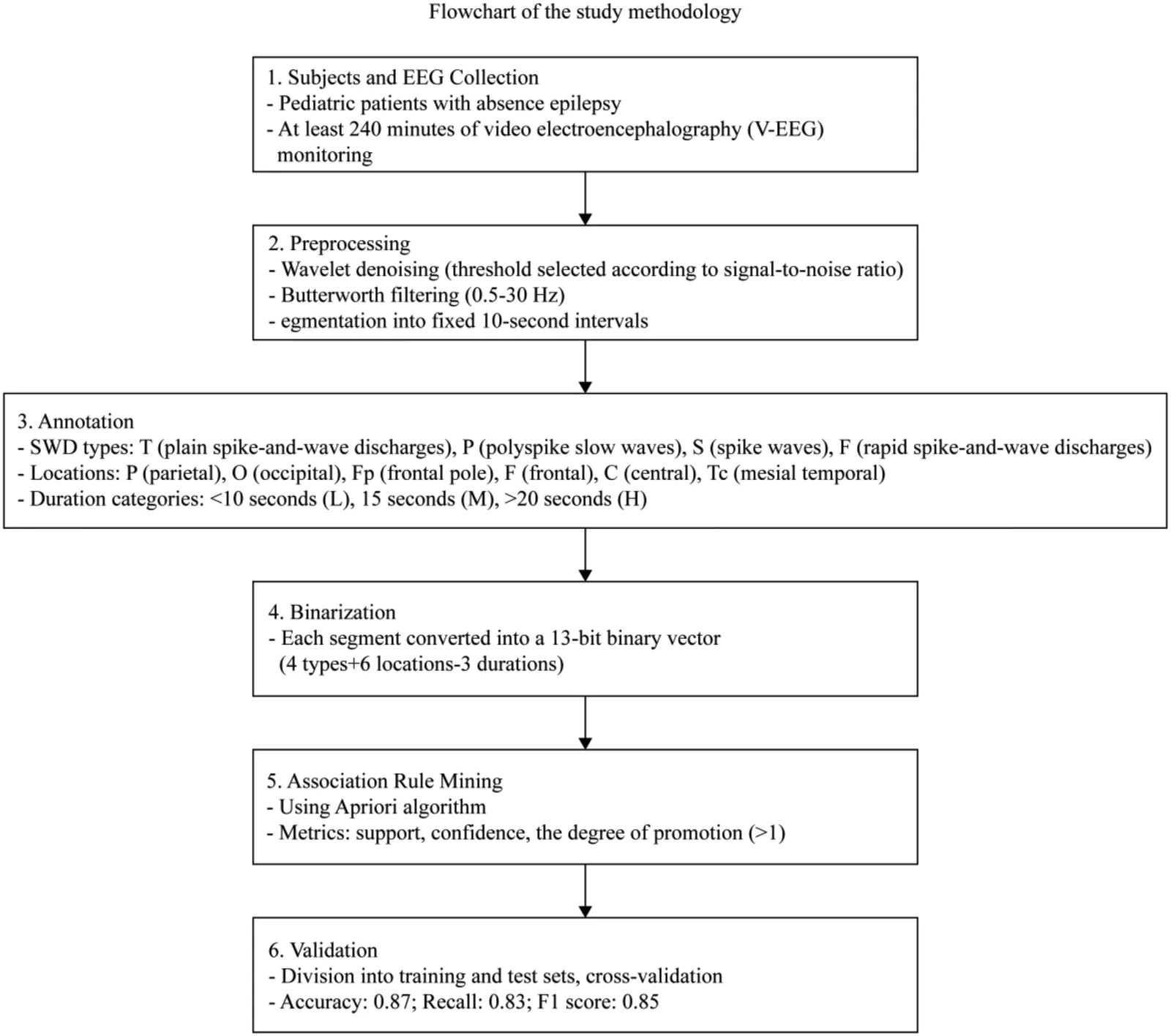
Flowchart of the study methodology. EEG = electroencephalography, SWD = spike-and-wave discharge, V-EEG = video-electroencephalography.

We evaluated the internal stability of the derived rules using 5-fold cross-validation. The dataset was split at the patient level to avoid leakage of multiple segments from the same individual across folds; in each fold, 80% of patients formed the training set and 20% formed the test set. Association rules were mined on the training set. For performance evaluation, we focused on rules whose consequent was the seizure-duration category (L/M/H). For each test transaction, the predicted duration class was determined by the applicable rule with the highest confidence, with ties resolved by higher lift. If no rule was applicable, the majority duration class from the training fold was assigned. Accuracy was defined as the proportion of correctly predicted duration classes. Recall and F1-score were computed per class and macro-averaged across the 3 duration categories. The reported accuracy, recall, and F1-score are the mean values across the 5 folds: accuracy 0.87, recall 0.83, and F1-score 0.85. This cross-validation procedure should be interpreted as internal validation within the available single-center dataset and does not substitute for independent external validation in a separate cohort (Fig. 1).

## 3. Results

### 3.1. *Characteristics of the subject* (Table 1)

The focus of this investigation was on EEG data obtained from pediatric patients diagnosed at our institution between January 2015 and June 2023. During that time, the initial EEG monitoring revealed synchronized symmetrical 3 Hz SWDs, which confirmed the diagnosis of AE.

**Table 1:** Baseline population table.

Hallmark	Descriptive
Distinguishing between the sexes	Male: 41.83%, Female: 58.17%
Age at debut	Under 12 yr, mainly 50–110 mo of age
Average age	93 mo
Course of disease	1 wk to 2 yr
Seizure duration	3–5 s 10 cases, 5–10 s 72 cases, 10–20 s 62 cases, 20–60 s 8 cases, 60–180 s 1 case
EEG monitoring time	4 h
Methods of EEG monitoring	See Methods
Diagnostic criteria	Classification of epilepsy syndromes according to the International League Against Epilepsy 2022

EEG = electroencephalography.

### 3.2. Visualization of data

This analysis examined association rules linking abnormal EEG discharge morphology, scalp distribution, and absence seizure duration. All key findings were interpreted using 3 quantitative metrics: support, confidence, and lift. Support reflects the prevalence of a rule in the transaction dataset, confidence reflects the conditional probability of the consequent given the antecedent, and lift > 1 indicates positive co-occurrence beyond chance. The main association rules and their corresponding metrics are summarized in Table 2.

**Table 2:** Consolidated summary of significant association rules (support, confidence, lift) shown in Figures 2 to 6.

Category	Association rule (antecedent → consequent)	Support	Confidence	Lift
Wave type–wave type	{P} → {SWD}	0.517	1.000	1.034
Wave type–wave type	{SWD} → {P}	0.517	0.534	1.034
Wave type–wave type	{spikes} → {SWD}	0.510	0.975	1.012
Wave type–wave type	{SWD} → {spikes}	0.510	0.527	1.008
Location–location	{MT} → {C}	0.601	0.968	1.528
Location–location	{C} → {MT}	0.588	0.968	1.528
Location–location	{F} → {C}	0.601	0.949	1.500
Location–location	{C} → {F}	0.313	0.949	1.496
Location–location	{Fp} → {C}	0.601	0.939	1.483
Location–location	{C} → {Fp}	0.601	0.949	1.481
Location–location	{Fp} → {F}	0.629	0.741	1.536
Location–location	{F} → {Fp}	0.627	0.740	1.545
Location–location	{Fp, F} → {C}	0.601	0.956	1.517
Location–location	{Fp, C} → {F}	0.601	0.996	1.569
Location–location	{F, C} → {Fp}	0.601	0.956	1.567
Location–location	{Fp} → {F, C}	0.601	0.996	1.569
Location–location	{F} → {Fp, C}	0.604	0.937	1.567
Location–location	{C} → {Fp, F}	0.604	0.937	1.569
Location–location	{Fp, generalized} → {F}	0.605	0.738	1.545
Location–location	{F, generalized} → {Fp}	0.605	0.738	1.545
Location–location	{Fp} → {F, generalized}	0.605	0.738	1.545
Location–location	{F} → {Fp, generalized}	0.605	0.956	1.544
Wave type–location	{F} → {SWD, Fp}	0.622	0.981	1.545
Wave type–location	{F} → {SWD, generalized, Fp}	0.601	0.948	1.544
Wave type–location	{Fp} → {SWD, F}	0.622	0.969	1.545
Wave type–location	{Fp} → {SWD, generalized, F}	0.601	0.939	1.545
Wave type–location	{generalized, F} → {SWD, Fp}	0.601	0.978	1.544
Wave type–location	{generalized, Fp} → {SWD, F}	0.601	0.969	1.543
Wave type–location	{SWD, F} → {Fp}	0.622	0.990	1.545
Wave type–location	{SWD, F} → {generalized, Fp}	0.601	0.960	1.544
Wave type–location	{SWD, Fp} → {F}	0.622	0.981	1.545
Wave type–location	{SWD, Fp} → {generalized, F}	0.604	0.948	1.544
Wave type–location	{SWD, generalized, F} → {Fp}	0.601	0.990	1.544
Wave type–location	{SWD, generalized, Fp} → {F}	0.601	0.978	1.544
Wave type–duration	{15.0 s} → {SWD}	0.400	0.985	1.021
Wave type–duration	{15.0 s} → {spikes}	0.229	0.565	1.072
Wave type–duration	{spikes} → {15.0 s}	0.229	0.438	1.081
Wave type–duration	{SWD, 15.0 s} → {P}	0.209	0.525	1.029
Wave type–duration	{P, 15.0 s} → {SWD}	0.209	1.000	1.034
Wave type–duration	{P, 7.5 s} → {SWD}	0.229	1.000	1.037
Wave type–duration	{SWD, 15.0 s} → {spikes}	0.229	0.574	1.089
Wave type–duration	{SWD, spikes} → {15.0 s}	0.229	0.449	1.106
Wave type–duration	{15.0 s, spikes} → {SWD}	0.229	1.000	1.048
Wave type–duration	{15.0 s} → {SWD, spikes}	0.229	0.565	1.097
Wave type–duration	{spikes, 7.5 s} → {SWD}	0.236	0.974	1.024
Location–duration	{F, 15.0 s} → {Fp}	0.289	1.000	1.561
Location–duration	{15.0 s, Fp} → {F}	0.289	0.978	1.545
Location–duration	{F} → {15.0 s, Fp}	0.289	0.455	1.542
Location–duration	{Fp} → {F, 15.0 s}	0.289	0.451	1.559
Location–duration	{F, 7.5 s} → {Fp}	0.262	0.976	1.529
Location–duration	{7.5 s, Fp} → {F}	0.262	0.976	1.537
Location–duration	{F} → {7.5 s, Fp}	0.262	0.414	1.539
Location–duration	{Fp} → {F, 7.5 s}	0.262	0.410	1.525

Duration values (15.0, 7.5) are in seconds.

Fp = frontal pole; F = frontal; C = central; MT = mesial temporal; SWD = spike-and-wave discharge; P = polyspike-and-wave.

**Figure 2.**
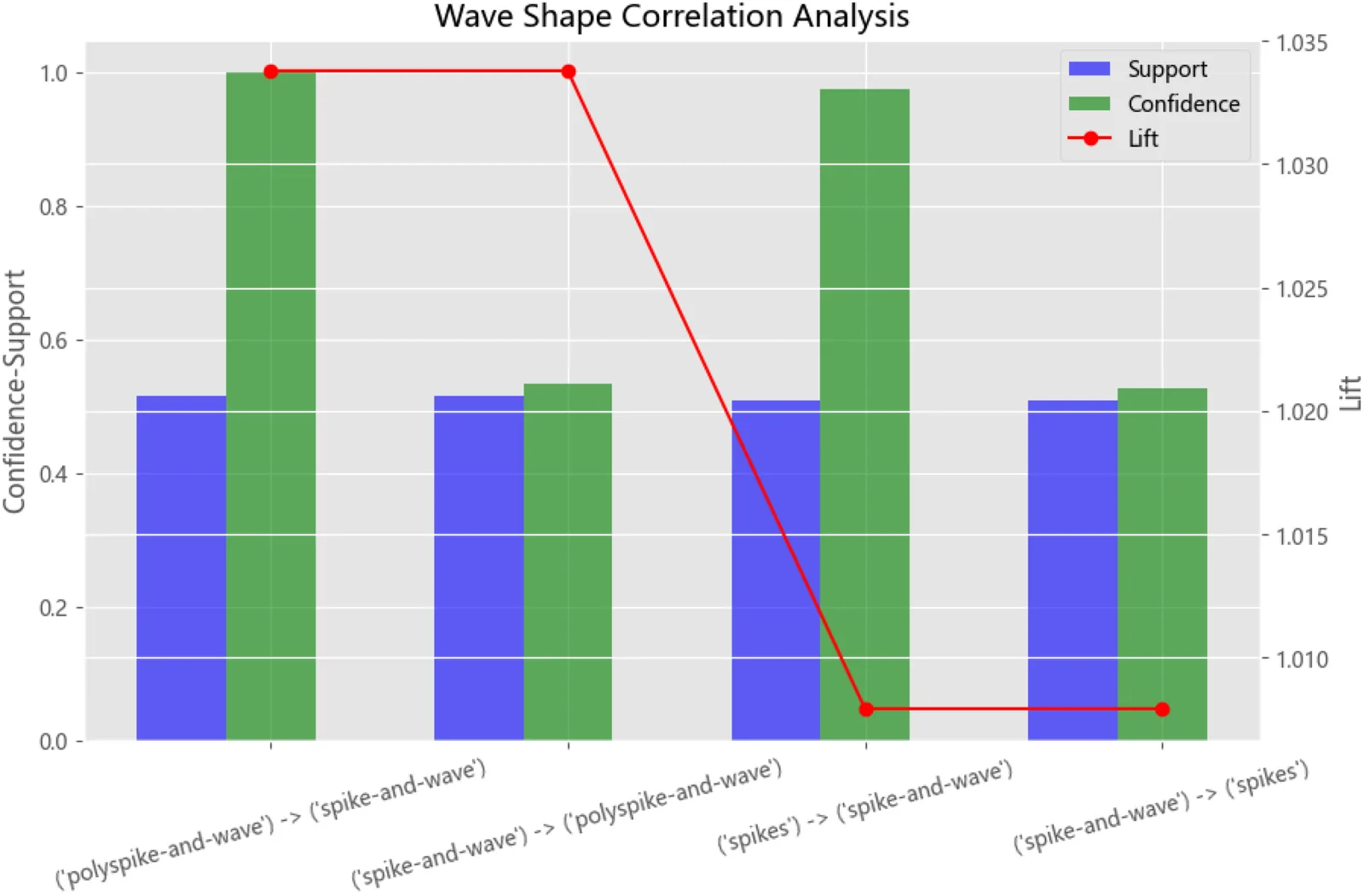
Abnormal EEG discharge morphologies. Representative EEG traces illustrating the waveform categories analyzed in this study. EEG = electroencephalography.

In the analysis of abnormal EEG discharge morphologies, we quantified asymmetric but consistent associations among major waveform categories (Table 2; Fig. 2). Specifically, polyspike-and-wave (P) → spike-and-wave discharge (SWD) showed support = 0.517, confidence = 1.000, and lift = 1.034, whereas SWD → P showed support = 0.517, confidence = 0.534, and lift = 1.034.

Similarly, spikes → SWD showed support = 0.510, confidence = 0.975, and lift = 1.012, while SWD → spikes showed support = 0.510, confidence = 0.527, and lift = 1.008. Full metrics for all significant wave-type rules are provided in Table 2 (Fig. 2).

**Figure 3.**
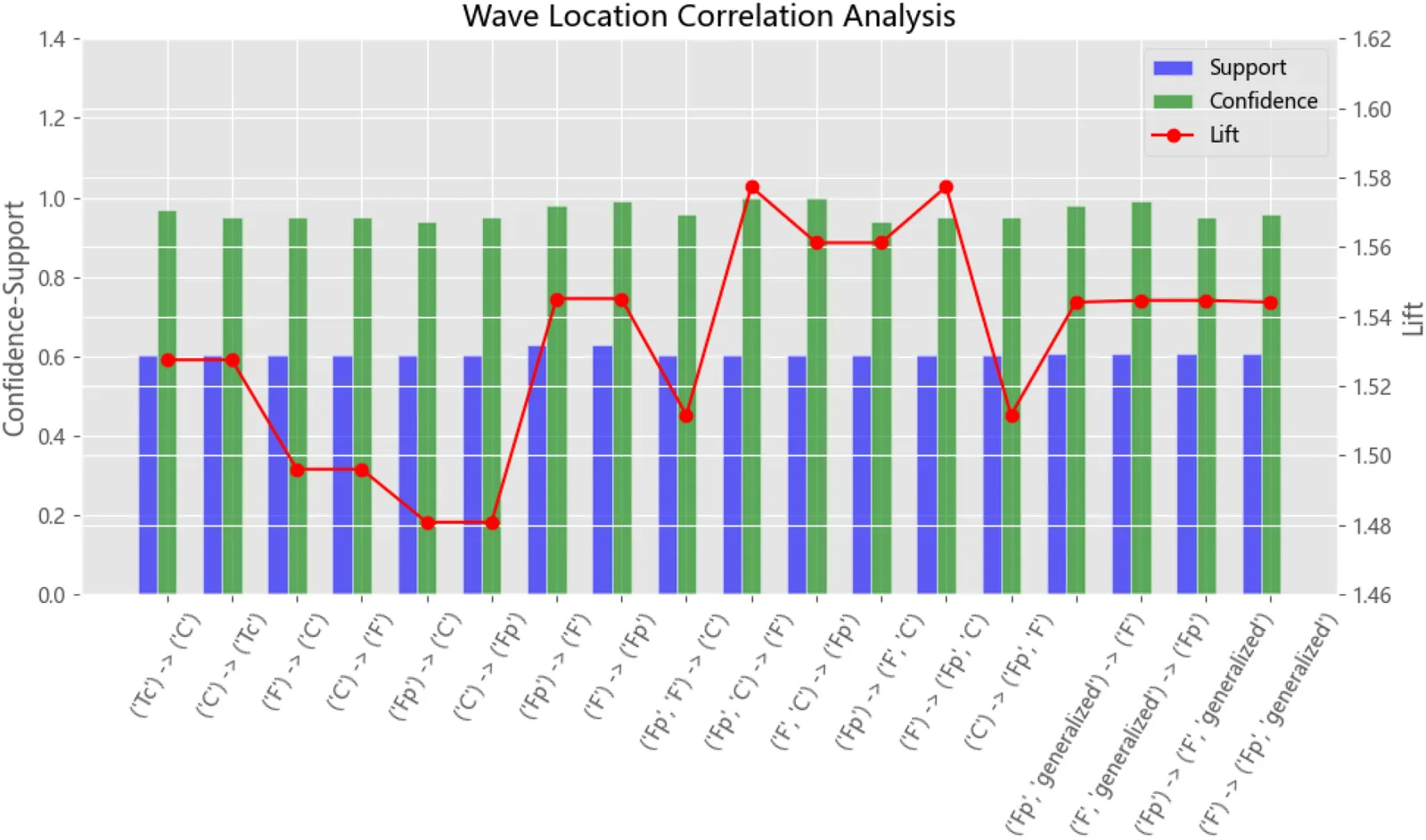
Scalp distribution of abnormal EEG discharges. The figure highlights the predominant EEG regions/channels involved across recordings. EEG = electroencephalography.

Location-level association rules were quantitatively supported by support values of approximately 0.588 to 0.601, confidence values of 0.939 to 0.968, and lift values of 1.483 to 1.528 for the main central-region rules (Table 2; Fig. 3). Specifically, the MT region and central region showed bidirectional co-occurrence (MT → C: support = 0.601, confidence = 0.968, lift = 1.528; C → MT: support = 0.588, confidence = 0.968, lift = 1.528). Frontal-to-central and frontal pole-to-central rules were also observed (F → C: support = 0.601, confidence = 0.949, lift = 1.500; Fp → C: support = 0.601, confidence = 0.939, lift = 1.483).

Frontal and frontal pole involvement showed frequent above-chance co-occurrence, as reflected by support values of 0.627 to 0.629 and lift values of 1.536 to 1.545 (Fp → F: support = 0.629, confidence = 0.741, lift = 1.536; F → Fp: support = 0.627, confidence = 0.740, lift = 1.545). In combination analyses, the {Fp, F} → {C} rule showed support = 0.601, confidence = 0.956, and lift = 1.517. All location-level rules and their metrics are summarized in Table 2 (Fig. 3).

**Figure 4.**
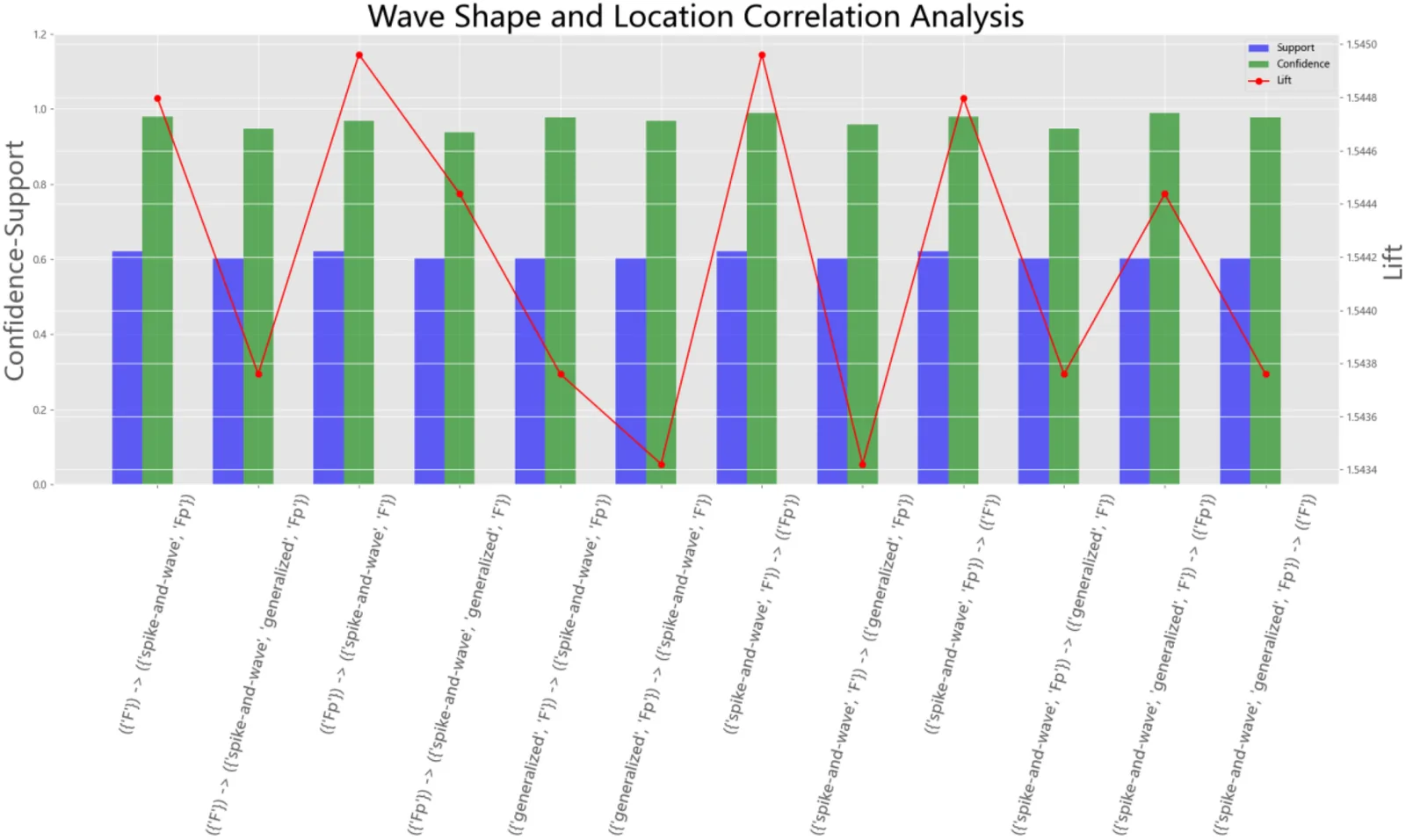
Associations between abnormal EEG discharge morphology and scalp region. EEG = electroencephalography.

Associations between EEG discharge morphology and scalp region were supported by high confidence values for SWD-related frontal/frontopolar rules (Table 2; Fig. 4). Specifically, F → {SWD, Fp} showed support = 0.622, confidence = 0.981, and lift = 1.545, while Fp → {SWD, F} showed support = 0.622, confidence = 0.969, and lift = 1.545. Conversely, the {SWD, F} → {Fp} rule showed support = 0.622, confidence = 0.990, and lift = 1.545.

At the transaction level, frontal/frontopolar rules involving concurrent SWD and another scalp region had support values of 0.622 and confidence values of 0.969 to 0.990, with lift values of 1.545. These metrics indicate an above-chance co-occurrence pattern between SWD-related morphology and frontal/frontopolar involvement, supporting the internal consistency of the identified rules (Fig. 4).

**Figure 5.**
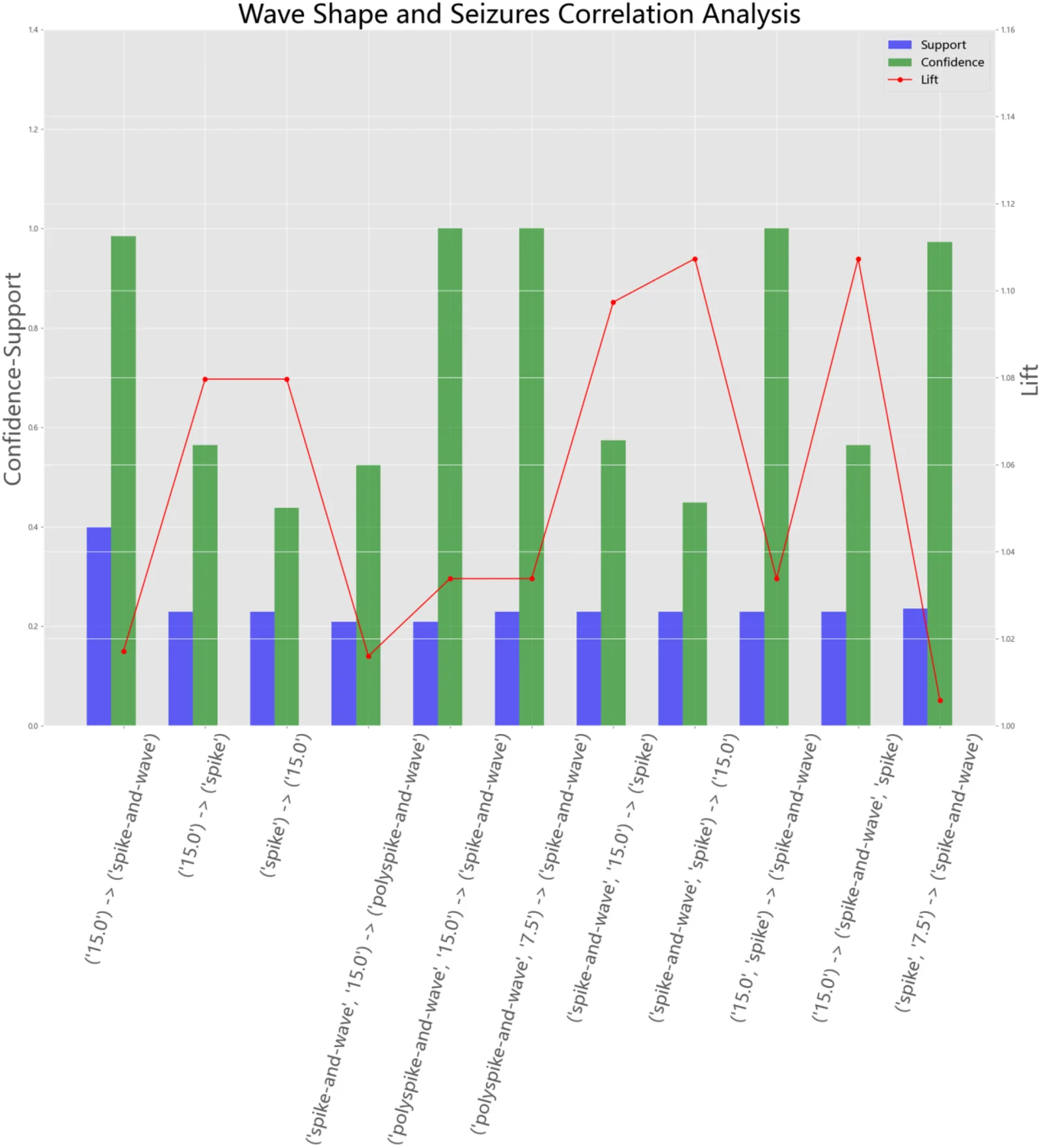
Associations between abnormal EEG discharge morphology and absence seizure duration. EEG = electroencephalography.

Analysis of the relationship between abnormal wave types and the duration of absence seizures identified multiple quantified association patterns (Table 2; Fig. 5). For 15.0-seconds seizures, {15.0 s} → {P} showed support = 0.400, confidence = 0.985, lift = 1.021, and {15.0 s} → {spikes} showed support = 0.229, confidence = 0.565, lift = 1.072. In the same duration stratum, co-occurrence within SWD segments was captured by {SWD, 15.0 s} → {P} (support = 0.209, confidence = 0.525, lift = 1.029).

At 7.5 seconds, P remained fully coupled with SWD ({P, 7.5 s} → {SWD}: support = 0.229, confidence = 1.000, lift = 1.037), and spikes at 7.5 seconds were also strongly linked to SWD ({spikes, 7.5 s} → {SWD}: support = 0.236, confidence = 0.974, lift = 1.024). Conversely, the presence of both SWD and spikes increased the probability of a 15.0-seconds seizure ({SWD, spikes} → {15.0 s}: support = 0.229, confidence = 0.449, lift = 1.106). Full metrics for all wave type–duration rules are provided in Table 2.

**Figure 6.**
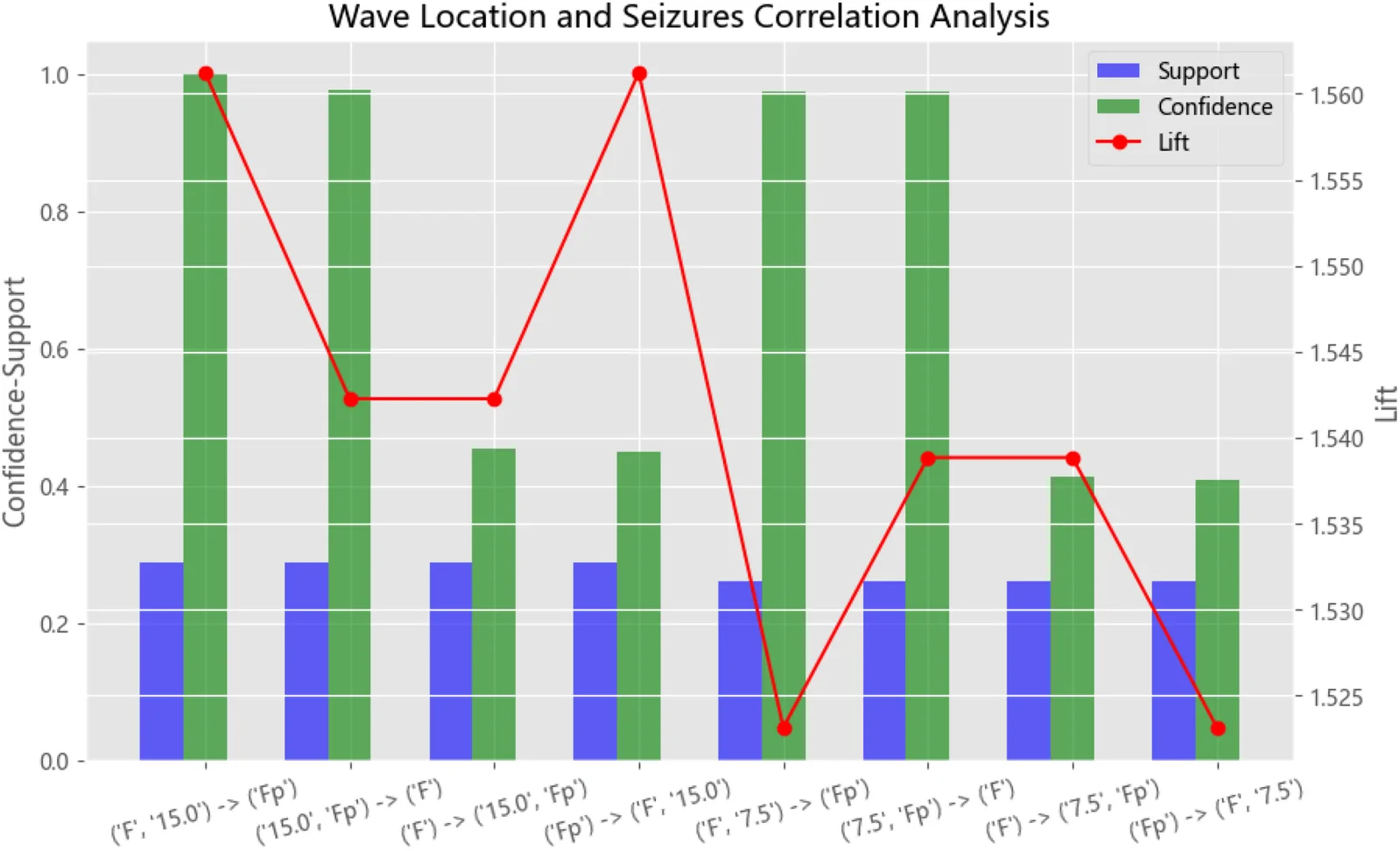
Associations between scalp distribution of abnormal EEG discharges and absence seizure duration. EEG = electroencephalography.

Correlation analysis between abnormal wave locations and absence seizure duration revealed consistent quantified patterns (Table 2; Fig. 6). At 15.0 seconds, frontal involvement almost always co-occurred with the frontal pole ({F, 15.0 s} → {Fp}: support = 0.289, confidence = 1.000, lift = 1.561), and the reverse rule remained high ({15.0 s, Fp} → {F}: support = 0.289, confidence = 0.978, lift = 1.545). Similar coupling was observed at 7.5 seconds ({F, 7.5 s} → {Fp}: support = 0.262, confidence = 0.976, lift = 1.529).

Collectively, frontal–frontal pole co-occurrence was observed across duration categories, with lift values ranging from 1.529 to 1.561 for the main duration-related rules ({F, 15.0 s} → {Fp}: support = 0.289, confidence = 1.000, lift = 1.561; {15.s, Fp} → {F}: support = 0.289, confidence = 0.978, lift = 1.545; {F, 7.5 s} → {Fp}: support = 0.262, confidence = 0.976, lift = 1.529). These findings indicate a reproducible scalp-level association pattern between frontal/frontopolar involvement and seizure-duration categories, rather than a causal relationship.

## 4. Discussion

Using ARM to analyze EEG data from children with AE, this study identified interpretable association rules linking abnormal EEG discharge morphology, scalp distribution, and absence seizure duration. Several rules showed consistent co-occurrence patterns, particularly involving frontal/frontopolar and central/MT regions. Importantly, these rules should not be interpreted as evidence that specific EEG morphologies or scalp regions cause longer seizure duration, greater seizure occurrence, or network propagation. Rather, they provide a structured summary of co-occurring EEG features, supported by the reported support, confidence, and lift values. Any implication regarding network-level synchronization should be regarded as hypothesis-generating rather than confirmatory. Given the single-center design, relatively small sample size, and lack of an independent validation cohort, the identified rules should be interpreted as internally derived co-occurrence patterns rather than externally validated clinical decision rules. Although 5-fold cross-validation suggested internal stability within the available dataset, it cannot determine whether these rules would remain stable across different institutions, EEG acquisition settings, patient populations, or labeling practices.

### 4.1. Associations between absence seizure duration and abnormal EEG discharge morphology

We identified association rules indicating that segments in the 10–20-seconds duration category, represented by the 15.0-seconds label in the rule output, were more likely to co-occur with spike-and-wave or polyspike-and-wave features. These findings suggest that absence seizure duration may co-occur with specific EEG discharge morphologies within the present dataset. However, these rule-based associations should be interpreted as descriptive co-occurrence patterns rather than causal or mechanistic relationships.^[16]^

These findings are partly consistent with previous studies. SWDs are characteristic EEG manifestations of AE, and SWD frequency or morphology may be related to seizure duration in some cohorts.^[17,18]^ Our association rules linking SWD-related morphologies with the 10 to 20-seconds duration category are therefore compatible with prior evidence, although direct comparison should be made cautiously because our analysis was based on discretized duration categories and co-occurrence rules rather than continuous event-level measures.

Some prior studies have suggested that rapid SWDs may be associated with shorter seizure durations.^[19,20]^ This differs from our rule-based findings, likely because prior studies often evaluated the relationship between continuous SWD frequency and seizure duration, whereas our study examined co-occurrence patterns involving predefined EEG morphology and duration categories. Differences in the definition of rapid SWD, age range, treatment exposure, baseline seizure burden, and analytic strategy may also contribute to the apparent inconsistency.

### 4.2. Associations between scalp distribution of abnormal EEG discharges and absence seizure patterns

We identified frequent co-occurrence between frontal and frontal pole involvement in the analyzed EEG segments. Rather than indicating a direct increase in seizure frequency, this rule should be interpreted as a scalp-level topographic pattern associated with absence seizure-related EEG activity.^[21,22]^ Previous studies have reported focal or frontal EEG features in patients with absence seizures and have linked frontal cortical regions to broader epileptic network activity.^[23,24]^ In this context, our frontal–frontopolar association is best interpreted as a reproducible EEG co-occurrence pattern rather than evidence of a causal relationship between frontal activity and seizure occurrence.

From a neurobiological perspective, repeated co-occurrence of frontal (F) and frontal pole (Fp) activity on scalp EEG may reflect consistent engagement of prefrontal cortical nodes within the broader corticothalamic networks implicated in absence seizures. Prefrontal regions interact with thalamic nuclei and large-scale control/attention systems, and transient synchronization across these circuits may facilitate the initiation or maintenance of generalized spike-and-wave activity, which could manifest as stable F–Fp coupling at the scalp level. At the same time, scalp EEG has limited spatial specificity; adjacency of F and Fp leads, volume conduction, and montage-dependent effects may also contribute to apparent co-activation. Therefore, we interpret the F–Fp association as a scalp-level co-occurrence pattern that is compatible with, but does not confirm, network-level synchronization. Source-level confirmation will require dedicated validation in future studies.

Some previous studies have emphasized temporal-region epileptiform activity or reported relationships between interictal spikes and seizure occurrence.^[25,26]^ Our rule set did not identify temporal-region features as dominant association rules for absence seizure duration or seizure-related EEG patterns. This difference should not be interpreted as a direct contradiction because ARM retains only patterns that meet predefined support, confidence, and lift thresholds. Potential explanations include differences in how “temporal” activity is defined (MT vs lateral temporal scalp leads), differences in montage and electrode coverage, and heterogeneity in patient populations and comorbid EEG patterns. Seizure frequency is also susceptible to clinical factors (e.g., medication status and disease stage) that were not explicitly modeled in our framework. Finally, ARM retains patterns that meet the chosen thresholds; less prevalent temporal patterns may not emerge as prominent rules even if clinically meaningful in a subset of patients.

### 4.3. Analysis and comparison with prior literature

Some of our findings are consistent with prior EEG studies, whereas others differ. Rather than interpreting these differences as direct contradictions, they should be considered in light of differences in cohort composition, single-center recruitment, EEG labeling schemes, segment selection, duration categorization, and ARM thresholds. The single-center recruitment from a specialized pediatric epilepsy clinic may have introduced center-specific influences, including referral patterns and EEG interpretation practices, which could affect the observed co-occurrence structure. Moreover, because ARM captures frequent co-occurrence patterns among predefined items, less common but clinically meaningful EEG patterns may not emerge as dominant association rules. Potential explanations for these inconsistencies include differences in ARM algorithms, data preprocessing, sample size, and uncommon combinations of EEG discharge morphologies and scalp locations in our dataset, all of which merit further investigation. In particular, differences in patient composition (syndrome subtype distribution, age, and medication exposure), definitions and labeling of waveform categories (e.g., frequency-defined rapid SWD vs morphology-based labels), segment selection rules, and duration discretization can all alter the co-occurrence structure captured by ARM. Accordingly, some divergences may reflect methodological and definitional heterogeneity rather than true biological contradiction. In addition, because clinical variables such as age, medication status, disease duration, seizure burden, and disease severity were not incorporated into the ARM framework, the observed rules may partly reflect unmeasured clinical heterogeneity. Thus, the current analysis should be interpreted as an exploratory EEG-pattern analysis rather than a clinically adjusted prediction model.

### 4.4. Comparison with alternative analytical approaches

Several analytical approaches can be used to study EEG data, and each has a different methodological purpose. Conventional time-frequency methods, including Fourier- and wavelet-based analyses, are useful for characterizing spectral composition, temporal evolution, and frequency-domain properties of EEG signals.^[27-30]^ These methods preserve more continuous signal information and are particularly suitable for studying oscillatory dynamics, discharge frequency, and time-varying EEG changes. However, their outputs are not always directly expressed as clinically interpretable combinations of waveform morphology, scalp distribution, and seizure-duration categories.

Machine learning methods, including supervised classification models, can capture complex nonlinear patterns and may improve predictive performance when sufficiently large, well-labeled, and externally validated datasets are available. Nevertheless, many machine learning models require larger sample sizes, careful feature engineering, and independent validation, and their outputs may be less transparent for clinicians if interpretability methods are not incorporated.

In contrast, ARM provides a transparent rule-based framework for identifying frequent co-occurrence patterns among predefined clinical EEG items. In the present study, ARM was not intended to replace time-frequency analysis or machine learning, nor was it designed to reconstruct the full neurophysiological complexity of EEG signals. Rather, it was used as an exploratory and interpretable method to summarize clinically labeled relationships among EEG discharge morphology, scalp distribution, and absence seizure-duration categories. Therefore, the methodological contribution of this study lies in demonstrating how ARM can complement conventional EEG analysis by generating clinically readable co-occurrence rules that may guide future hypothesis-driven and externally validated studies.

This methodological choice also explains an important trade-off of the present study. Transforming EEG morphology, scalp distribution, and seizure duration into predefined binary items made the data suitable for ARM and improved the interpretability of the resulting rules. However, this transformation inevitably simplified the underlying neurophysiological data. Information on waveform amplitude, exact discharge frequency, temporal evolution, propagation sequence, and continuous seizure duration was not retained in the binary transaction matrix. Therefore, the resulting rules should be viewed as simplified, clinically interpretable summaries rather than comprehensive representations of the full EEG signal. Future studies may integrate ARM with time-frequency features or machine learning models to evaluate whether rule-based co-occurrence patterns remain informative when richer quantitative EEG features are included.

## 5. Limitations

Several limitations should be acknowledged. First, this was a single-center retrospective study with a relatively small sample size. Although all patients underwent standardized video-EEG monitoring within the same institution, the resulting association rules may reflect center-specific referral patterns, EEG acquisition procedures, patient composition, and expert labeling practices. Therefore, the findings should be interpreted as internally derived EEG co-occurrence patterns rather than externally validated rules.

Second, EEG feature labeling was based on expert manual annotation. Although labeling was performed using a predefined coding scheme and was checked for internal consistency before binarization, formal inter-observer agreement statistics, such as Cohen’s κ, were not available because of the retrospective design. This may have introduced observer-dependent variability. Future studies should include independent dual labeling, consensus adjudication, and quantitative inter-observer agreement assessment.

Third, although 5-fold cross-validation was performed at the patient level to reduce data leakage and assess internal stability, this procedure cannot establish external generalizability. Cross-validation reuses partitions from the same source population and is therefore still influenced by the same center-specific referral patterns, EEG acquisition procedures, labeling conventions, and patient characteristics. Because no independent external validation cohort was available in the current retrospective analysis, the robustness of the identified rules across other clinical settings remains uncertain. Independent validation in larger multicenter cohorts is required before these rules can be applied more broadly.

Fourth, the current study did not include a formal empirical comparison with machine learning models or quantitative time-frequency EEG analysis. Therefore, we cannot determine whether ARM provides superior predictive performance or signal-level characterization compared with these approaches. The value of ARM in this study lies mainly in its interpretability and its ability to summarize clinically labeled EEG co-occurrence patterns. Future studies should compare ARM with machine learning and time-frequency methods using larger datasets, external validation cohorts, and standardized performance metrics.

Fifth, binarization of EEG features and discretization of seizure duration simplified complex neurophysiological information. Although this transformation was necessary for transaction-based ARM and improved interpretability, it reduced information on continuous EEG characteristics, including amplitude, exact frequency, temporal evolution, propagation sequence, and continuous seizure duration. Therefore, the identified rules should be interpreted as simplified co-occurrence summaries rather than comprehensive representations of EEG dynamics.

Sixth, potentially relevant clinical covariates, including age, sex, medication status, disease duration, seizure burden, disease severity, and treatment exposure, were not incorporated into the ARM framework. These factors may influence EEG morphology, scalp distribution, and seizure duration and may therefore confound some observed associations. As a result, the current findings should not be regarded as clinically adjusted prediction rules.

Finally, because ARM identifies co-occurrence patterns rather than causal relationships, the observed associations do not establish that a specific EEG morphology or scalp region causes a particular seizure-duration category. Similarly, because scalp EEG has limited spatial resolution, the topographic rules identified in this study should be interpreted as scalp-level co-occurrence patterns rather than source-localized neural activity.

Future studies should include larger multicenter cohorts, harmonized EEG acquisition and labeling procedures, independent inter-observer reliability assessment, additional clinical variables, and external validation to confirm the stability, reproducibility, and clinical applicability of the identified association rules.

## 6. Conclusion

In summary, ARM applied to single-center pediatric EEG data identified interpretable co-occurrence patterns linking abnormal EEG discharge morphology, scalp distribution, and seizure duration in AE, with principal rules showing support values of 0.262 to 0.629, confidence values of 0.741 to 1.000, and lift values of 1.012 to 1.561. ARM may complement conventional time-frequency EEG analysis and machine learning approaches by providing transparent, clinically readable co-occurrence rules based on predefined EEG features. However, because the analysis was based on expert manual labeling, binarized EEG features, discretized seizure-duration categories, an unadjusted ARM framework, and internal validation only, the findings should be interpreted cautiously. Larger multicenter studies with independent labeling reliability assessment, additional clinical variables, external validation, and comparison with alternative analytical approaches are needed to determine whether these rules are reproducible and generalizable across clinical settings.

## Acknowledgments

We would like to extend our special thanks to Jie Li, Hengxing Zhang, Xiaojin Long, and particularly to Hao Dong Zhu for their contributions to this research. Mr. Hao Dong Zhu provided invaluable technical support and professional advice in the research design, data analysis, and experimental process. Furthermore, we are grateful to all the staff at Kunming Children’s Hospital, as well as the patients and their families who participated in this study. Their support and involvement were essential to the completion of this work.

## Author contributions

**Conceptualization:** Xiaomei Liu.

**Investigation:** Lei Li.

**Methodology:** Lingxiang Ao, Lei Li, Kai Liu.

**Project administration:** Lijun Li.

**Software:** Li Wang.

**Writing – original draft:** Lijun Li, Lingxiang Ao.

**Writing – review & editing:** Kai Liu, Xiaomei Liu.

## References

[1] Studer F Jarre G Pouyatos B. Aberrant neuronal connectivity in the cortex drives generation of seizures in rat absence epilepsy. Brain. 2022;145:1978–91. 10.1093/brain/awab438 35141747

[2] Wirrell EC Grossardt BR Wong-Kisiel LC Nickels KC. Incidence and classification of new-onset epilepsy and epilepsy syndromes in children in Olmsted County, Minnesota from 1980 to 2004: a population-based study. Epilepsy Res. 2011;95:110–8. 10.1016/j.eplepsyres.2011.03.009 PMC3260338 21482075

[3] Fastenau PS Shen J Dunn DW. Academic underachievement among children with epilepsy: proportion exceeding psychometric criteria for learning disability and associated risk factors. J Learn Disabil. 2008;41:195–207. 10.1177/0022219408317548 PMC2748109 18434287

[4] Yayici Köken O Şekeroğlu B Şanlidağ B Sari Yanartaş M Yilmaz A. Focality in childhood absence epilepsy. Neurol Res. 2024;46:626–33. 10.1080/01616412.2024.2339114 38643974

[5] Tatum WO. Handbook of EEG Interpretation. Demos Medical Publishing; 2014

[6] Kumar A Lyzhko E Hamid L Srivastav A Stephani U Japaridze N. Neuronal networks underlying ictal and subclinical discharges in childhood absence epilepsy. J Neurol. 2023;270:1402–15. 10.1007/s00415-022-11462-8 PMC9971098 36370186

[7] Han X Jiang F Needleman J Zhou H Yao C Tang Y-L. Comorbidity combinations in schizophrenia inpatients and their associations with service utilization: a medical record-based analysis using association rule mining. Asian J Psychiatr. 2022;67:102927. 10.1016/j.ajp.2021.102927 34847493

[8] Kalgotra P Sharda R. BIARAM: a process for analyzing correlated brain regions using association rule mining. Comput Methods Programs Biomed. 2018;162:99–108. 10.1016/j.cmpb.2018.05.001 29903499

[9] Wu M Zhang X Yu H Pan H. Mining association rules between pressure injury risk factors in adult inpatients based on the apriori algorithm. Comput Inform Nurs. 2025;43:e01348. 10.1097/CIN.0000000000001348 40638211

[10] Vespa PM Olson DM John S. Evaluating the clinical impact of rapid response electroencephalography: the DECIDE multicenter prospective observational clinical study. Crit Care Med. 2020;48:1249–57. 10.1097/CCM.0000000000004428 PMC7735649 32618687

[11] Fisher RS Cross JH French JA. Operational classification of seizure types by the international league against epilepsy: position paper of the ILAE commission for classification and terminology. Epilepsia. 2017;58:522–30. 10.1111/epi.13670 28276060

[12] Chaddad A Wu Y Kateb R Bouridane A. Electroencephalography signal processing: a comprehensive review and analysis of methods and techniques. Sensors (Basel). 2023;23:6434. 10.3390/s23146434 PMC10385593 37514728

[13] Exarchos TP Tzallas AT Fotiadis DI Konitsiotis S Giannopoulos S. EEG transient event detection and classification using association rules. IEEE Trans Inf Technol Biomed. 2006;10:451–7. 10.1109/titb.2006.872067 16871711

[14] Ramezankhani A Pournik O Shahrabi J Azizi F Hadaegh F. An application of association rule mining to extract risk pattern for type 2 diabetes using tehran lipid and glucose study database. Int J Endocrinol Metab. 2015;13:e25389. 10.5812/ijem.25389 PMC4393501 25926855

[15] Phukhachee T Maneewongvatana S Chaiyanan C Iramina K Kaewkamnerdpong B. Identifying the effect of cognitive motivation with the method based on temporal association rule mining concept. Sensors (Basel). 2024;24:2857. 10.3390/s24092857 PMC11086084 38732962

[16] Meritam Larsen P Wüstenhagen S Terney D Gardella E Aurlien H Beniczky S. Duration of epileptic seizure types: a data-driven approach. Epilepsia. 2023;64:469–78. 10.1111/epi.17492 PMC10107943 36597206

[17] Glaba P Latka M Krause MJ. EEG phase synchronization during absence seizures. Front Neuroinform. 2023;17:1169584. 10.3389/fninf.2023.1169584 PMC10317177 37404335

[18] Datta AN Crawford J Wallbank L Wong PKH. Outcome of absence epilepsy with onset at 8-11 years of age: watershed ages when syndromes overlap. J Child Neurol. 2023;38:505–12. 10.1177/08830738231188397 PMC10493039 37461321

[19] Garzon P Lemelle L Auvin S. Childhood absence epilepsy: an update. Arch Pediatrie. 2016;23:1176–83. 10.1016/j.arcped.2016.08.005 27683026

[20] Holmes GL McKeever M Adamson M. Absence seizures in children: clinical and electroencephalographic features. Ann Neurol. 1987;21:268–73. 10.1002/ana.410210308 3111345

[21] Matur Z Baykan B Bebek N Gürses C Altindağ E Gökyiğit A. The evaluation of interictal focal EEG findings in adult patients with absence seizures. Seizure. 2009;18:352–8. 10.1016/j.seizure.2009.01.007 19213578

[22] Rodin E Litzinger M Thompson J. Complexity of focal spikes suggests relative epileptogenicity. Epilepsia. 1995;36:1078–83. 10.1111/j.1528-1157.1995.tb00465.x 7588451

[23] Ibrahim GM Cassel D Morgan BR. Resilience of developing brain networks to interictal epileptiform discharges is associated with cognitive outcome. Brain. 2014;137(Pt 10):2690–702. 10.1093/brain/awu214 PMC4163036 25104094

[24] Tenney JR Williamson BJ Kadis DS. Cross-frequency coupling in childhood absence epilepsy. Brain Connect. 2022;12:489–96. 10.1089/brain.2021.0119 34405685

[25] Ren L Kucewicz MT Cimbalnik J. Gamma oscillations precede interictal epileptiform spikes in the seizure onset zone. Neurology. 2015;84:602–8. 10.1212/WNL.0000000000001234 PMC4335986 25589669

[26] Karoly PJ Freestone DR Boston R. Interictal spikes and epileptic seizures: their relationship and underlying rhythmicity. Brain. 2016;139(Pt 4):1066–78. 10.1093/brain/aww019 26912639

[27] Puliafito V Vergura S Carpentieri M. Fourier, wavelet, and hilbert-huang. transforms for studying electrical users in the time and frequency domain. Energies. 2017;10:188.

[28] Chong KL Lai SH El-Shafie A. Wavelet transform based method for river stream flow time series frequency analysis and assessment in tropical environment. Water Resour Manag. 2019;33:2015–32.

[29] Akin M. Comparison of wavelet transform and FFT methods in the analysis of EEG signals. J Med Syst. 2002;26:241–7. 10.1023/a:1015075101937 12018610

[30] Kiymik MK Güler I Dizibüyük A Akin M. Comparison of STFT and wavelet transform methods in determining epileptic seizure activity in EEG signals for real-time application. Comput Biol Med. 2005;35:603–16. 10.1016/j.compbiomed.2004.05.001 15809098

